# Influences of habitat and seasonal changes on gonadal maturation of *Echinometra mathaei* (Echinodermata: Echinoidea) and *Tridacna squamosa* (Mollusca: Bivalvia) in the Red Sea, Egypt

**DOI:** 10.1007/s10661-023-11713-9

**Published:** 2023-08-24

**Authors:** Samaa G. El-Sokkary, Khaleid F. Abd El-Wakeil, Ahmad H. Obuid-Allah, Mohsen Y. Omer

**Affiliations:** 1 Zoology and Entomology Department, Faculty of Science, Assiut University, Assiut, Egypt; 2 National Institute of Oceanography and Fisheries (NIOF), Red Sea Branch, Hurghada, Egypt

**Keywords:** Active reproduction, Marine ecosystem, Gametes, Histologic analysis, Temperature

## Abstract

The present work is aimed at investigating the effects of seasonal changes and habitat conditions on reproductive state and gonadal development of two macrobenthic fauna. *Echinometra mathaei* and *Tridacna squamosa* were collected seasonally (summer and winter) in July 2019 and January 2020 from three sites along the Red Sea coast, Egypt: Hamraween, Sedy Malek, and Porto Ghalb. Sexual differences and gonadal maturation in the two species were determined by performing biopsies of their gonads followed by morphologic analyses. Hematoxylin and eosin–stained gonadal sections were examined and analyzed. In *E. mathaei*, reproductive behavior was more active in the summer than in the winter; in *T. squamosa*, reproductive behavior was active almost all the year, especially during the winter. The reproductive activity and gonadal maturation of both species were affected by environmental factors. The results indicated that temperature is a vital factor affecting the reproductive activity of both species. This study concluded that temperature fluctuations may pose significant challenges to coastal marine ecosystems.

**Supplementary Information:**

The online version contains supplementary material available at 10.1007/s10661-023-11713-9.

## Introduction

Reproduction and growth, as life-history strategies and responses of animals to environmental conditions, are related to the aging process in the animal kingdom (Blier et al., 2017). The activities of marine benthic invertebrates have major biologic and geologic effects on a wide range of habitats in tropical and temperate environments (McClanahan & Muthiga, 2007). Changes in environmental factors coordinate reproduction of marine animals, which leads to seasonal reproductive cycles (Hasan, 2019; Mercier & Hamel, 2009). Studies of the reproduction of marine macrobenthos are important to determine their growth rate and the characteristics of the next generation. Mercier and Hamel (2009) stated that understanding the interaction of exogenous and endogenous factors on the efficiency of gametes at suitable times of the year is important not only from an ecologic viewpoint but also for fisheries and aquaculture programs, in addition to evaluating how populations may respond to and be affected by natural and anthropogenic disturbances (e.g., climate change, fisheries, and pollution).

Animals are valuable tools as bioindicators to monitor marine pollution and have been largely used to measure the quality of health of the coastal environment (Al-Howiti et al., 2020; Hamza-Chaffai, 2014). *Echinometra mathaei* (de Blainville, 1825) (Echinoidea) and *Tridacna squamosa* Lamarck, 1819 (Bivalvia) were chosen as models in the present study because of their abundance along the Red Sea coast (El-Sokkary et al., 2022; Mahdy et al., 2019) and their tolerance to various pollutants (Al-Howiti et al., 2020; Vahideh et al., 2012). Both animals have vital roles in marine ecosystems (e.g., they as filter, and detritus feeders clean the habitats as well as accelerate detritus decomposition. *E. mathaei* is the most abundant sea urchin in the world (Muthiga, 1996). Its flexibility in distribution, reproduction, and feeding allows it to adapt to variable environmental conditions (McClanahan & Muthiga, 2007). *E. mathaei* has important geologic and biologic effects on coral reefs, seagrass beds, and kelp forests (Muthiga, 1996).

*T. squamosa* is native to the Indo-Pacific region (Huelsken et al., 2013). It has a high growth rate and is able to tolerate a wide range of environmental conditions (Van Wynsberge et al., 2017). Studies in past decades focused on the reproduction of bivalves to improve management decisions (Hold et al., 2013), pest control (Ram et al., 1996), and aquaculture farming (Joaquim et al., 2008). Menoud et al. (2016) recommended studying the seasonal fluctuations of maturity stages in clams and their relationship with environmental factors, which are crucial for understanding the regeneration of wild clam populations. Few studies have considered the reproductive activity of giant clams (Mies & Sumida, 2012; Van Wynsberge et al., 2017).

Environmental degradation in the coastal zone has been further exacerbated by anthropogenic activity, climate change, and extreme behavior (Lu et al., 2018). According to Lu et al. (2018), the world’s seas are the main sink for the excess thermal energy arising from global warming. From 1950 to 2014, the surface sea temperature (SST) increased at a rate of roughly 0.114 °C each decade (Karl et al., 2015). Bronstein et al. (2016) mentioned that the future of animals that rely on seasonal cues to coordinate reproduction is uncertain as global warming and climate change progress even more quickly. Thus, comprehension of the mechanisms must be improved that this led to underlying and control this process in broadcast spawning species (Bronstein & Loya, 2015).

Research on distinguishing the seasonal fluctuations of maturity stages of benthic marine fauna and their relationship to environmental impacts is highly recommended. This will be critical for understanding the regeneration of wild populations and will provide fundamental information for future studies on these populations. The objectives of the present study were to describe the gametogenesis and gonadal conditions of *E. mathaei* and *T. squamosa* collected from different sites along the Red Sea coast during different seasons and to investigate the effects of environmental conditions on the reproductive state and gonadal development of these bioindicator species.

## Materials and methods

The climate in Egypt is characterized by hot summer and moderate winter (Domroes & EL-Tantawi, 2005); therefore, sampling was carried out in summer and winter to investigate seasonal effects. To conduct this study, three diverse locations with a variety of human activities were chosen. At the sites under investigation, the two chosen species are dominant. It may be easier to generalize the results to other species by using two distinct species that demonstrate changes in their reproductive behaviors in response to environmental conditions.

### Study sites and sampling groups

**Fig. 1:**
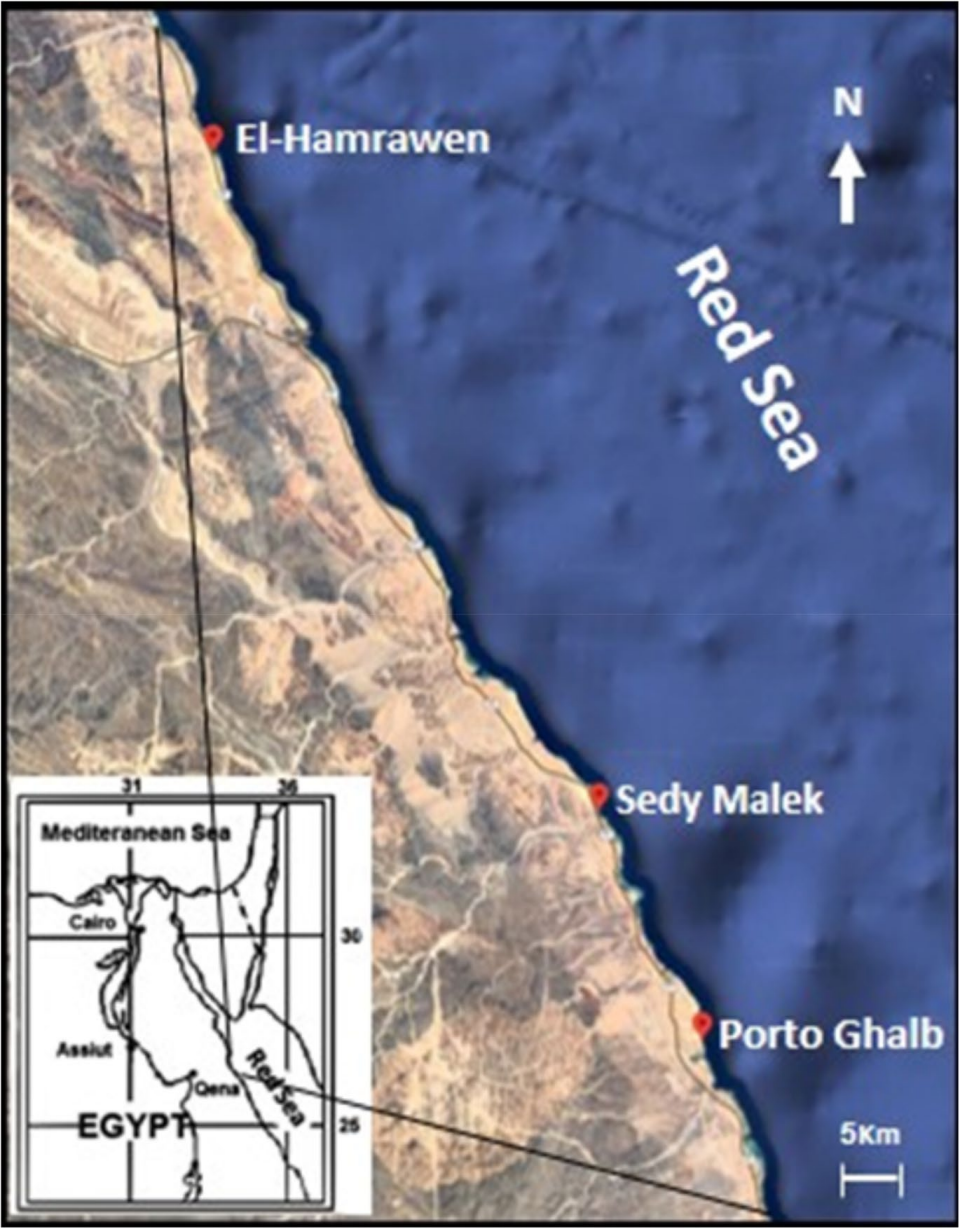
Map showing the location of the three investigated sites on the Red Sea coast of Egypt

El-Hamrawen, Sedy Malek, and Porto Ghalb on the Red Sea coast of Egypt were chosen as collecting sites (Fig. 1). El-Hamrawen is located at 26° 15′ 04.7″ N 34° 12′ 11.6″ E, about 120 km south of Hurghada, 60 km south of Safaga, and 20 km north of Al-Quseir, which contains the oldest and largest phosphate harbors on the Egyptian Red Sea coast. Sedy Malek is located at 25° 43′ 40.2″ N 34° 32′ 47.5″ E, about 50 km south of Al-Quseir. It is in front of a religious tourism area, where people go to visit Sheikh Malek’s shrine and their activities increase the organic stress at this site. Porto Ghalb is an integrated port and resort community located at 25° 32′ 44.9″ N 34° 38′ 26.1″ E, about 70 km north of Marsa Alam.

Animal samples were collected during the summer (July 2019) from El-Hamrawen and Sedy Malek and during the winter (January 2020) from all three sites. The samples were coded as sampling groups Hs, Ms, Hw, Mw, and Pw, referring to specimens collected during the summer at El-Hamrawen, during the summer at Sedy Malek, during the winter at El-Hamrawen, during the winter at Sedy Malek, and during the winter at Porto Ghalb, respectively.

### Collecting samples

*T. squamosa* and *E. mathaei* samples were collected by scuba diving and snorkeling in the depth reach to 5 m at the investigated sites. During sampling, the environmental variables air and water temperature (°C), water hydrogen ion concentration (WpH), salinity (ppt), dissolved oxygen (mg L^−1^), and conductivity (ms cm^−1^) were measured using Hydrolab (YSI Pro DSS Multi-Parameter Water Quality Meter). Sediment samples (about 1 kg/sample) were collected from the investigated sites, dried in direct sunlight after removing the existing biota (fauna and flora), and preserved for analysis of carbonate and organic matter contents. In the laboratory, sediment total organic matter content (TOC), carbonate content, and sediment grain size (three major groups: coarse sediment group (CSG) comprises fractions > 1 mm, medium sediment group (MSG) includes fractions between 0.250 and 1 mm, and fine sediment group (FSG) includes fractions < 0.0250 mm) were estimated according to the methods of Basaham and El-Sayed (1998), Brenner and Binford (1988), and Folk (1974), respectively.

### Animal treatment in the field

In the field, eight specimens of both *T. squamosa* and *E. mathaei* from each sampling group were weighed and dissected. The test diameter (the longest radius) for *E. mathaei* and the shell length of *T. squamosa*, were measured by a ruler. The gonads of each specimen were weighed to calculate the gonadosomatic index (GSI) as the ratio of gonad wet weight to flesh animal weight (Keshavarz et al., 2017; Menoud et al., 2016).

$$\mathrm{GSI}=\mathrm{GW}/\mathrm{AW}\;^\ast100$$

where GW = gonad weight in grams (g) and AW = animal weight in grams (g).

Because the gonadal tissue of *T. squamosa* cannot be easily separated from the digestive gland, the weighed part included both gonad and digestive gland. *T. squamosa* weight was measured without the byssus (Menoud et al., 2016). The gonad samples were fixed and preserved in 10% formalin until reached the laboratory for sectioning and histologic analysis.

### Histologic analysis

In the laboratory, for histologic examination, the gonad samples were transferred to 70% ethanol until further processing. The gonads were dehydrated, embedded in paraffin wax, and sectioned at 5- to 7-µm thickness. The sections were stained with hematoxylin and eosin (Gabe, 1976) and examined by light microscope (Olympus CHT, BX43F Tokyo163-0914 Japan). Three photographs at different locations from selected four sections per collected animals were taken by XCAM1080PHA camera (Tokyo, Japan). The ImageJ program was used to calculate the ratio of gametes to nutritive cells and ovum area for the taken photos (SM 1). For both *E. mathaei* and *T. squamosa*, the longest and shortest diameters of 15 oocytes were measured in each photograph to calculate the ovum area as oval shape. To define the reproductive stages of *E. mathaei* and *T. squamosa*, Siddique and Ayub (2019) and Menoud et al. (2016) were followed, respectively.

### Statistical analysis

Data summary and analysis were performed by IBM SPSS Statistics (Version 20) and Excel Office 2010. The independent-samples *t*-test was used to test differences in the mean GSI between the two studied animals. One-way analysis of variance was used to determine significant differences between sample groups. The Duncan test was used to detect different variances between means. The relationships between GSI and test diameter of *E. mathaei* and shell length *T. squamosa* were assessed by linear regression analysis. The PAST4 program was used to perform principal component analysis (PCA) of the mean values of environmental variables and investigated reproductive parameters after normalization (normalization resembles to the ratio of the difference between each data entry (*x*_*i*_) and the respective mean (*m*) and the standard deviation (sd) of a given variable (*x*_*i*_ − *m*/sd)).

## Results

Table 1 shows the mean GSI of *E. mathaei* and *T. squamosa* in different sample groups. The results indicated that the GSI of *T. squamosa* was higher than that of *E. mathaei* (*t* = 12.57, *p* < 0.001). The GSI of *E. mathaei* ranged between 3.05 and 6.56. The lowest value of GSI was recorded at Sedy Malek during the winter and was significantly different from that in the other samples (*F* = 4.104, *p* = 0.007). The GSI of *T. squamosa* ranged between 12.24 and 19.32. The lowest value was recorded during the summer in Hamraween (*F* = 2.894, *p* = 0.034). It is worth mentioning that the GSI was positively correlated with the test diameter of *E. mathaei* (*r*^2^ = 0.335) and negatively correlated with the shell length of *T. squamosa* (*r*^2^ = 0.833).

**Table 1:** Mean values of the gonadosomatic index (GSI) of *Echinometra mathaei* and *Tridacna squamosa* for different sample groups (*Hs*, El-Hamrawen samples during summer; *Ms*, Sedy Malek samples during summer; *Hw*, El-Hamrawen samples during winter; *Mw*, Sedy Malek samples during winter; *Pw*, Porto Ghalb samples during winter)

Sample groups	*E. mathaei*	*T. squamosa*	*t*	*p* value
	Mean ± Std.D	Mean ± Std.D		
Hs	6.56 ± 2.76**a**	12.24 ± 4.78**b**	**12.57**	** < 0.001**
Ms	4.78 ± 2.24**a**	14.29 ± 3.32**ab**		
Hw	5.96 ± 2.04**a**	16.93 ± 4.89**ab**		
Mw	3.05 ± 1.19**b**	17.93 ± 3.63**a**		
Pw	4.52 ± 1.88**a**	19.32 ± 8.56**a**		
*F*	**4.104**	**2.894**		
*p* value	**0.007**	**0.034**		

Similar characters indicate no significant difference. Values in bold indicate statistical results

**Fig. 2:**
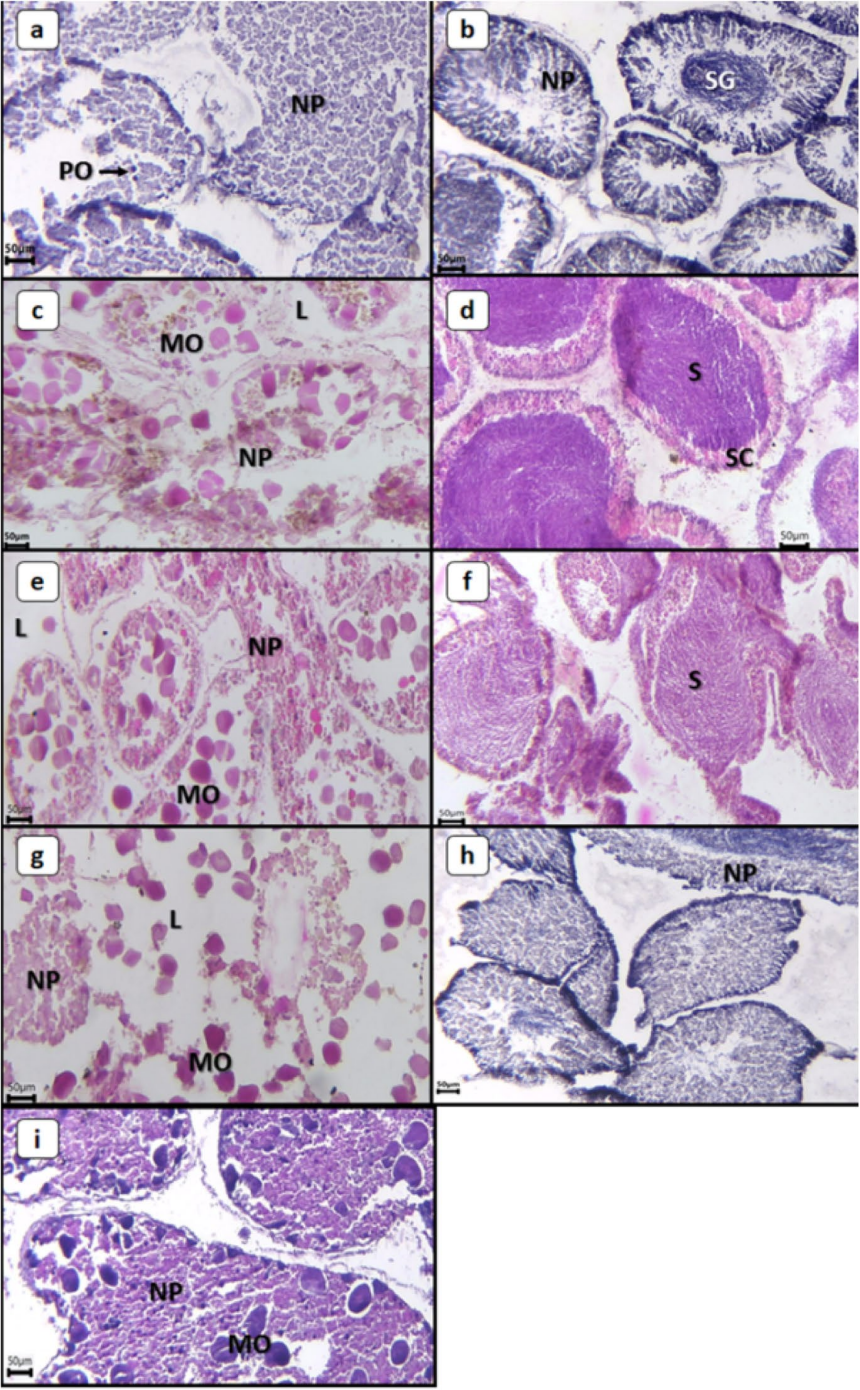
Photographs of reproductive stages of male and female gonads of *Echinometra mathaei*. **a** Immature/resting stage in female; **b** immature/resting stage in male; **c** premature stage in female; **d** premature stage in male; **e** mature stage in female; **f** mature stage in male; **g** partially spawned stage in female; **h** spent stage in male; **i** spent stage in female. L lumen, MO mature ovum, NP nutritive phagocytes, PO previtellogenic ovum, S sperm, SC spermatocytes, SG spermatogonia

The color of *E. mathaei* gonads was orange to light brown in both sexes and was generally darker in females than in males. Both sexes have several reproductive stages: immature/resting, premature, mature, partially spawned, and spent. In the present study, all these stages were observed in different seasons: premature, mature, and partially spawned in the summer and immature and spent in the winter (Fig. 2).

**Fig. 3:**
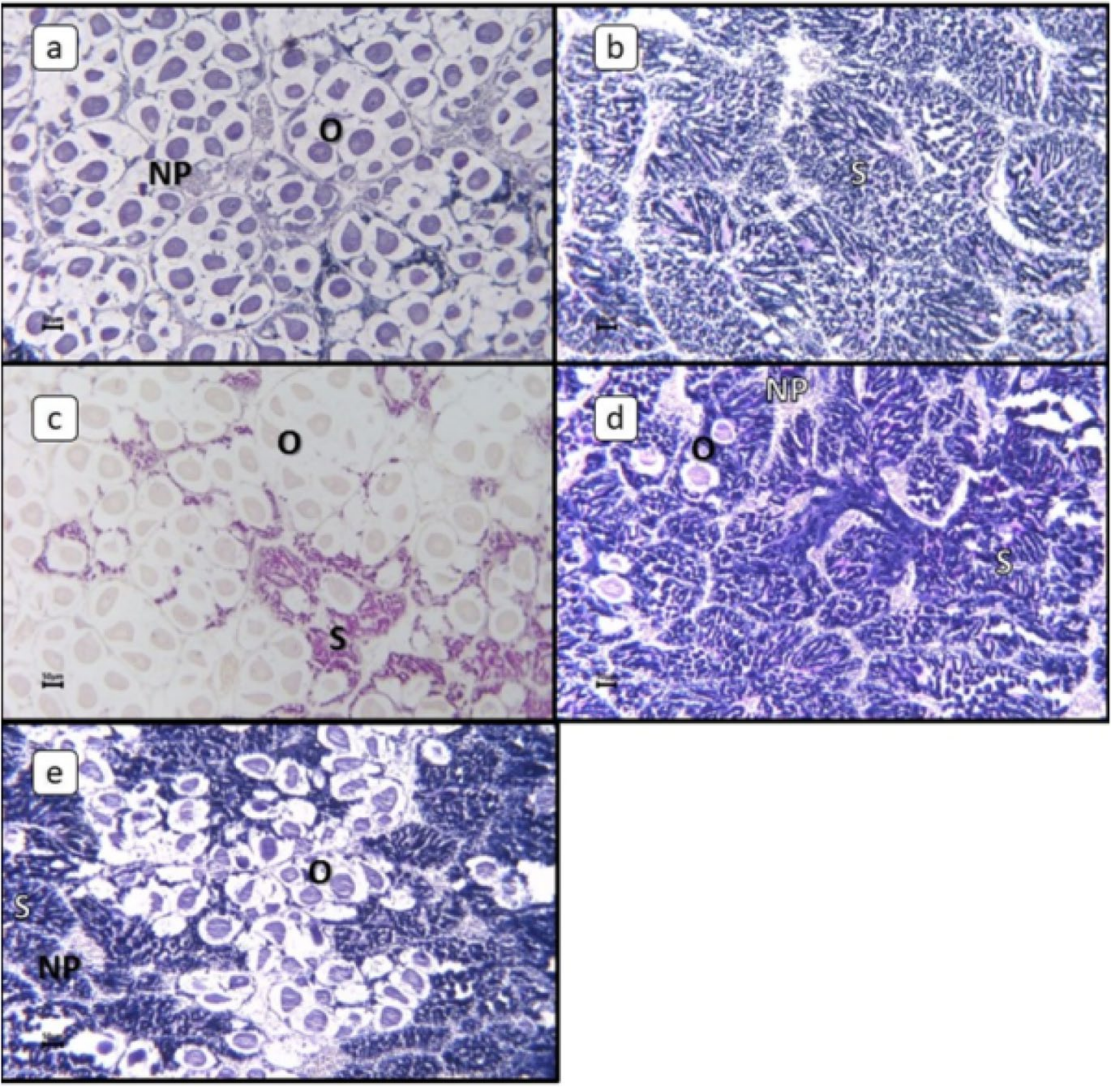
Photomicrographs of gonadal tissue of *Tridacna squamosa*. **a** Female; **b** male; **c** female dominant; **d** male dominant; **e** male and female. NP nutritive phagocytes, O ovum, S sperm

*T. squamosa* is hermaphroditic; when male gametes are more frequent than female gametes, it is termed male dominant, and when female gametes are more frequent than male gametes, it is termed female dominant. Some *T. squamosa* had only male or female gametes (Fig. 3). Mature and immature reproductive stages were observed in *T. squamosa*. In female *T. squamosa*, full maturation can be detected by the shape and size of the ovum. Mature ova are oval in shape.

**Fig. 4:**
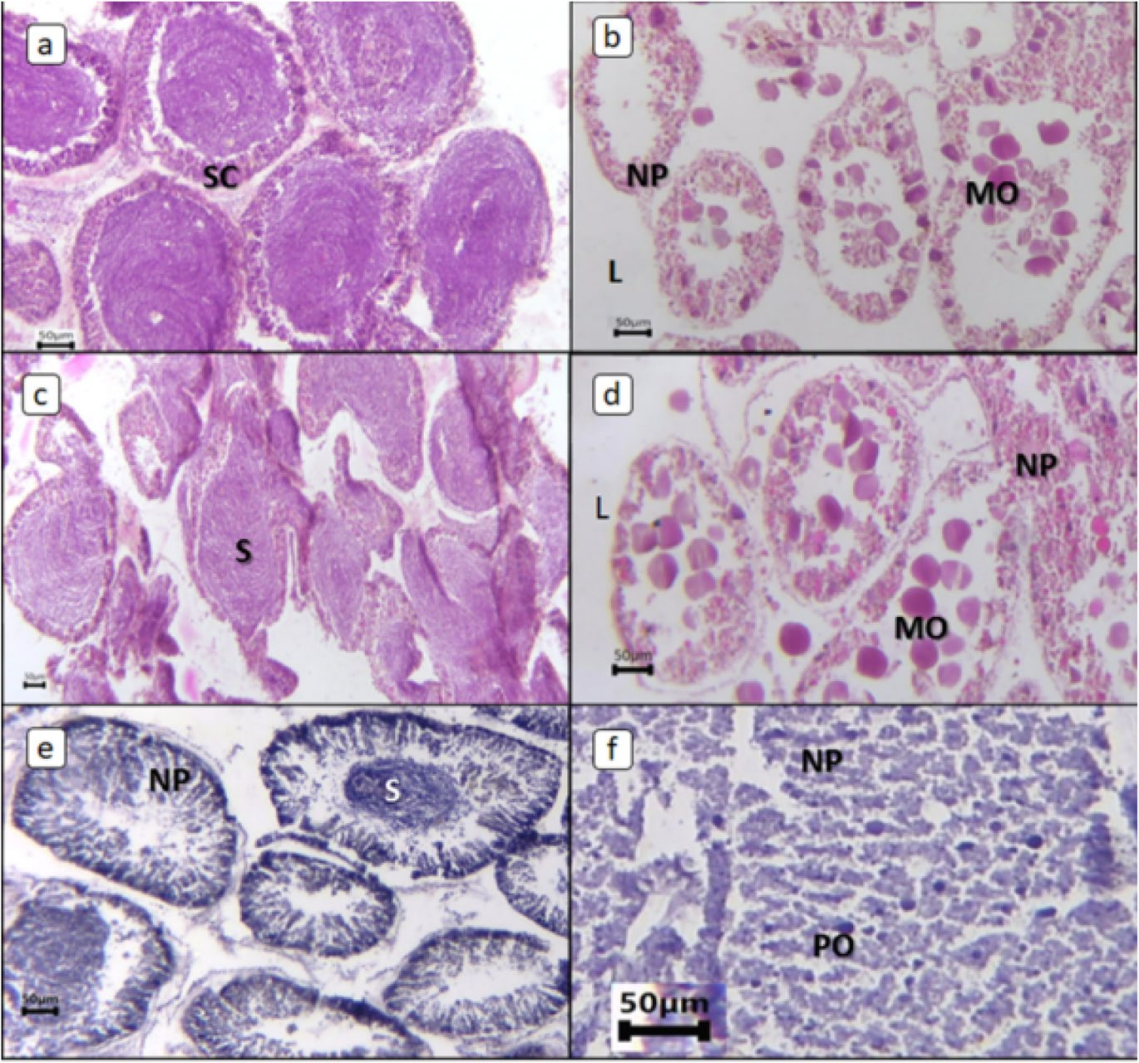
Photomicrographs of microscopical characteristics of male and female gonads of *Echinometra mathaei*. **a** Male; **b** female; **c** high level of gametes in male; **d** high level of gametes and large ovum size in female; **e** low level of gametes in male; **f** low level of gametes and small ovum size in female. L lumen, MO mature ovum, NP nutritive phagocytes, PO previtellogenic ovum, S sperm, SC spermatocytes

**Fig. 5:**
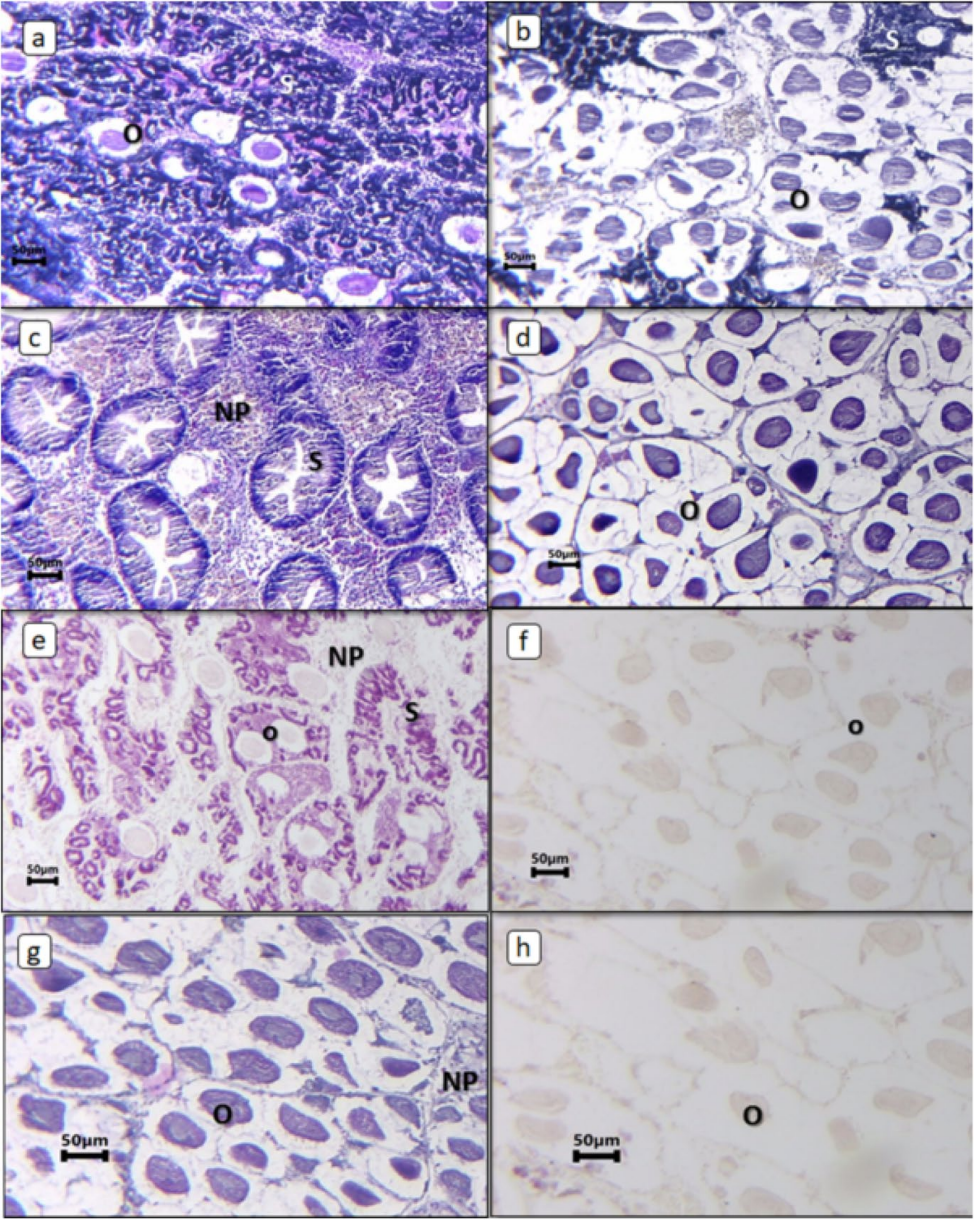
Photomicrographs of microscopical characteristics of *Tridacna squamosa*. **a** Male dominant; **b** female dominant; **c** high level of gametes in male; **d** high level of gametes in female; **e** low level of male gametes; **f** low level of female gametes; **g** high level of gametes and large ovum size; **h** low level of gametes and small ovum size. NP nutritive phagocytes, O ovum, S sperm

**Fig. 6:**
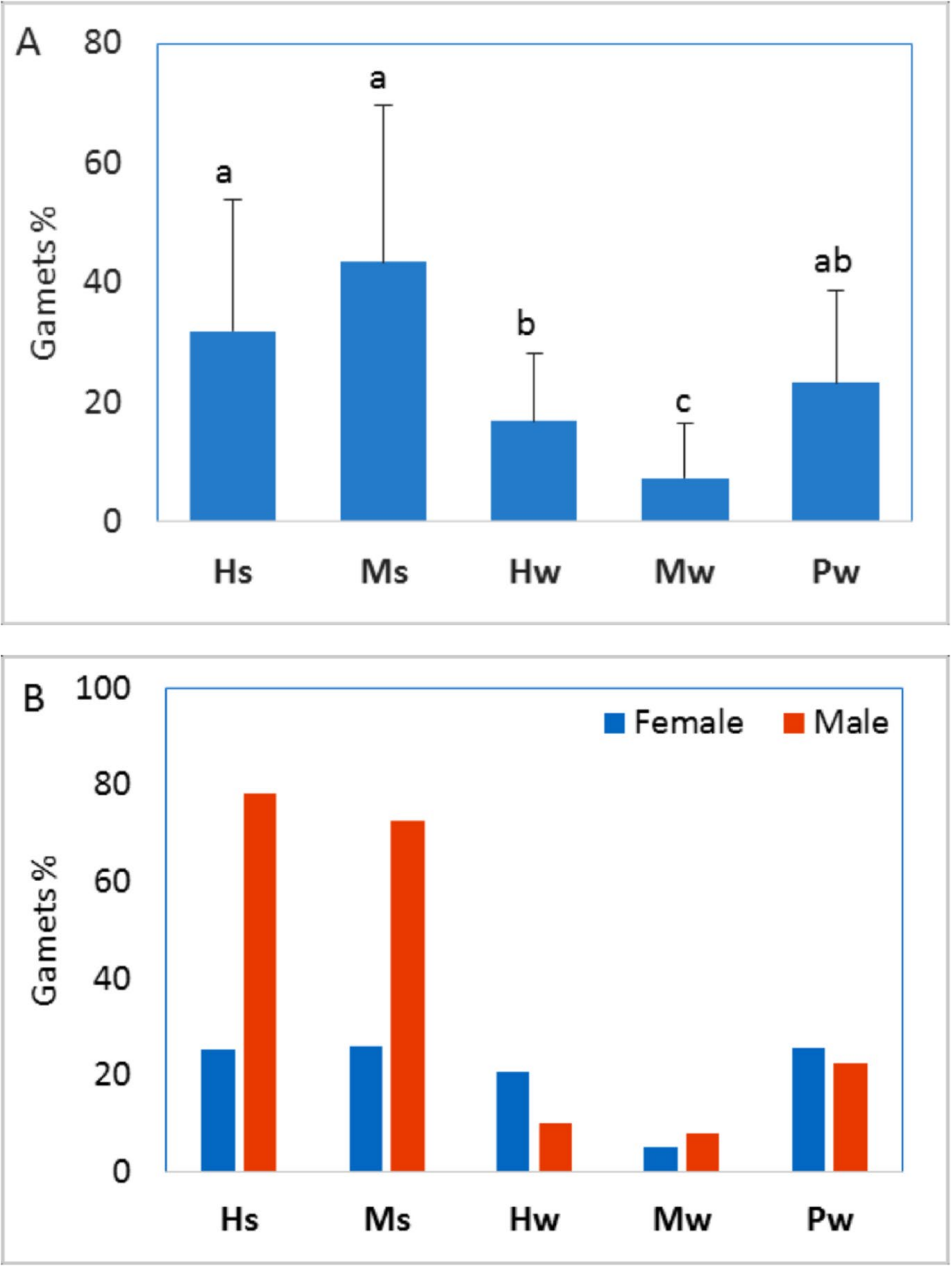
**A** Means and standard deviations of gamete ratio of *Echinometra mathaei*. **B** Means of gamete ratio for males and females for different sample groups (different characters indicate significant differences among sample groups)

**Fig. 7:**
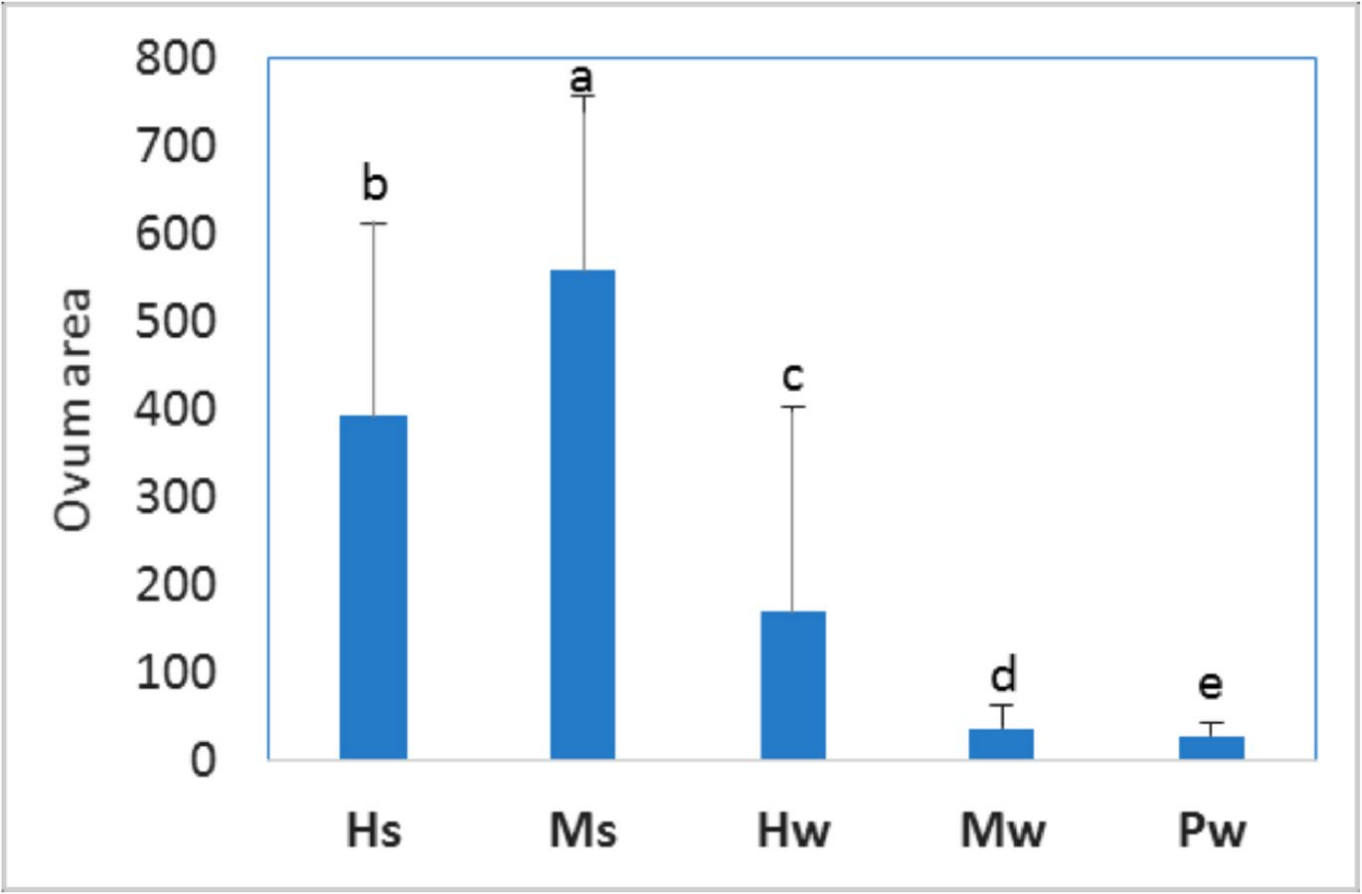
Means and standard deviations of ovum area (µm.^2^) for *Echinometra mathaei* collected from different sample groups (different characters indicate significant differences among sample groups)

Changes in histologic characteristics of the gonads among the studied samples are illustrated in Figs. 4 and 5 for *E. mathaei* and *T. squamosa*, respectively. In both males and females, the percentage of *E. mathaei* gametes was significantly higher during the summer than during the winter. The percentage of gametes was lowest during the winter in Sedy Malek (Figs. 4 and 6A); the percentage of male gametes was highest during the summer in Hamraween and Sedy Malek; and the percentages of female gametes and large ova were highest during the summer in Sedy Malek (Figs. 6B and 7).

**Fig. 8:**
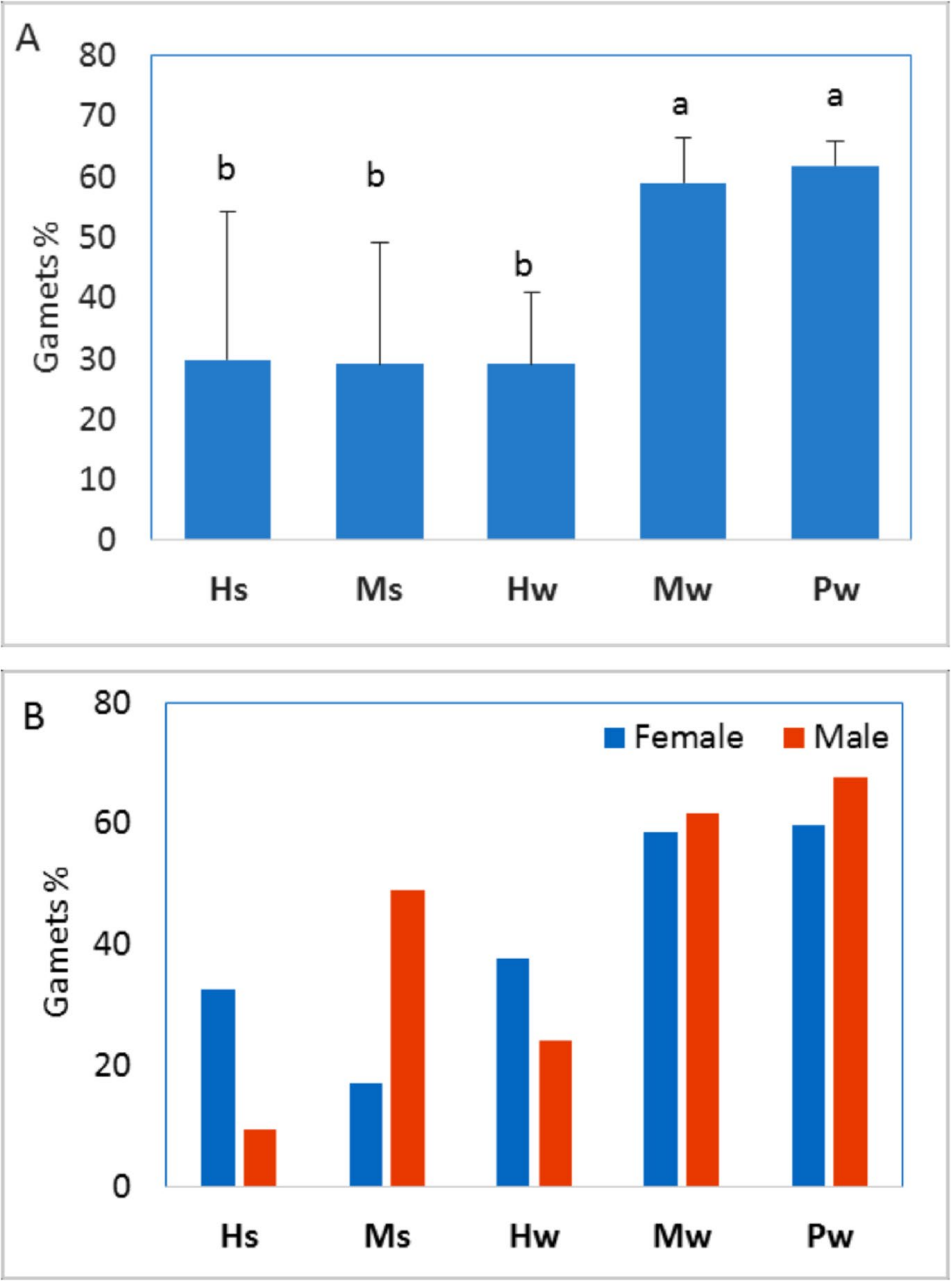
**A** Means and standard deviations of gamete ratio of *Tridacna squamosa*. **B** Means of gamete ratio for male and female gonads for different sample groups (different characters indicate significant differences among sample groups)

**Fig. 9:**
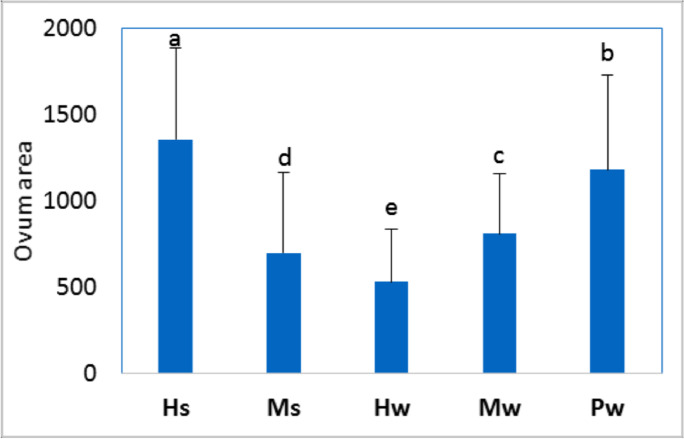
Means and standard deviations of ovum area (µm.^2^) for *Tridacna squamosa* collected from different sample groups (different characters indicate significant differences among sample groups)

*T. squamosa* showed an opposite trend to *E. mathaei*; the percentage of gametes was higher during the winter than during the summer. The percentage of gametes was highest during the winter in Porto Ghalb and lowest during the winter in Hamraween (Figs. 5 and 8A). Spawning females and male gametes of *T. squamosa* were recorded in both seasons, but more mature and productive individuals of this species were recorded during the winter (Figs. 5 and 8B). In Hamraween, large ova (1352.4 µm^2^) were detected during the summer, and small ova (527.7 µm^2^) were detected during the winter (Fig. 9).

Correlation coefficients (*r* values) for the associations between GSI, percentage of gametes, and ovum area of *E. mathaei* and *T. squamosa* with investigated environmental variables, and their significance are represented in Table 2. The results indicated influences of environmental variables on reproductive activity and gonadal maturation in both *E. mathaei* and *T. squamosa*.

**Table 2:** Correlation coefficients ( *r* values) for associations between percentage of gametes and ovum area of *Echinometra mathaei* and *Tridacna squamosa* with investigated environmental variables

Environmental variables	*Echinometra mathaei*						*Tridacna squamosa*					
	GSI		Gametes%		Ovum area		GSI		Gametes%		Ovum area	
	*r*	Sig	*r*	Sig	*r*	Sig	*r*	Sig	*r*	Sig	*r*	Sig
Airtemp	0.459	0.437	0.930^*^	0.022	0.931^*^	0.021	−0.856	0.064	−0.618	0.267	0.320	0.599
Wtemp	0.497	0.394	0.913^*^	0.030	0.935^*^	0.020	−.887^*^	0.045	−0.650	0.235	0.323	0.596
WpH	0.648	0.237	0.388	0.519	0.272	0.658	−0.579	0.306	−0.216	0.727	0.883^*^	0.047
Salinity	0.114	0.855	0.323	0.596	0.696	0.191	−0.603	0.281	−0.712	0.177	−0.576	0.309
DO	−0.355	0.558	−0.529	0.359	−0.848	0.069	.905^*^	0.034	0.768	0.129	0.100	0.873
TDS	−0.036	0.955	0.431	0.468	0.027	0.965	0.370	0.540	0.181	0.770	0.019	0.975
Cond	0.508	0.382	0.912^*^	0.031	0.962^**^	0.009	−.900^*^	0.038	−0.704	0.185	0.243	0.693
Carbonate	0.441	0.457	0.828	0.083	0.990^**^	0.001	−.901^*^	0.037	−0.797	0.106	−0.018	0.977
TOC	0.581	0.304	0.377	0.532	0.229	0.711	−0.523	0.366	−0.132	0.833	0.923^*^	0.026
CSG	0.501	0.390	−0.209	0.735	−0.264	0.668	−0.196	0.752	0.061	0.922	0.763	0.134
MSG	0.224	0.717	0.606	0.279	0.199	0.749	−0.079	0.899	0.137	0.826	0.684	0.203
FSG	−0.358	0.555	−0.463	0.432	−0.089	0.887	0.132	0.832	−0.140	0.822	−0.844	0.072

^**^Correlation is significant at the 0.01 level (2-tailed)

^*^Correlation is significant at the 0.05 level (2-tailed)

*Airtemp* air temperature, *Wtemp* water temperature, *WpH* water hydrogen ion concentration, *salinity* water salinity, *DO* dissolved oxygen, *Cond* conductivity, *carbonate* sediment carbonate content, *TOC* sediment total organic matter content, *CSG* coarse sediment group, *MSG* medium sediment group, *FSG* fine sediment group

**Fig. 10:**
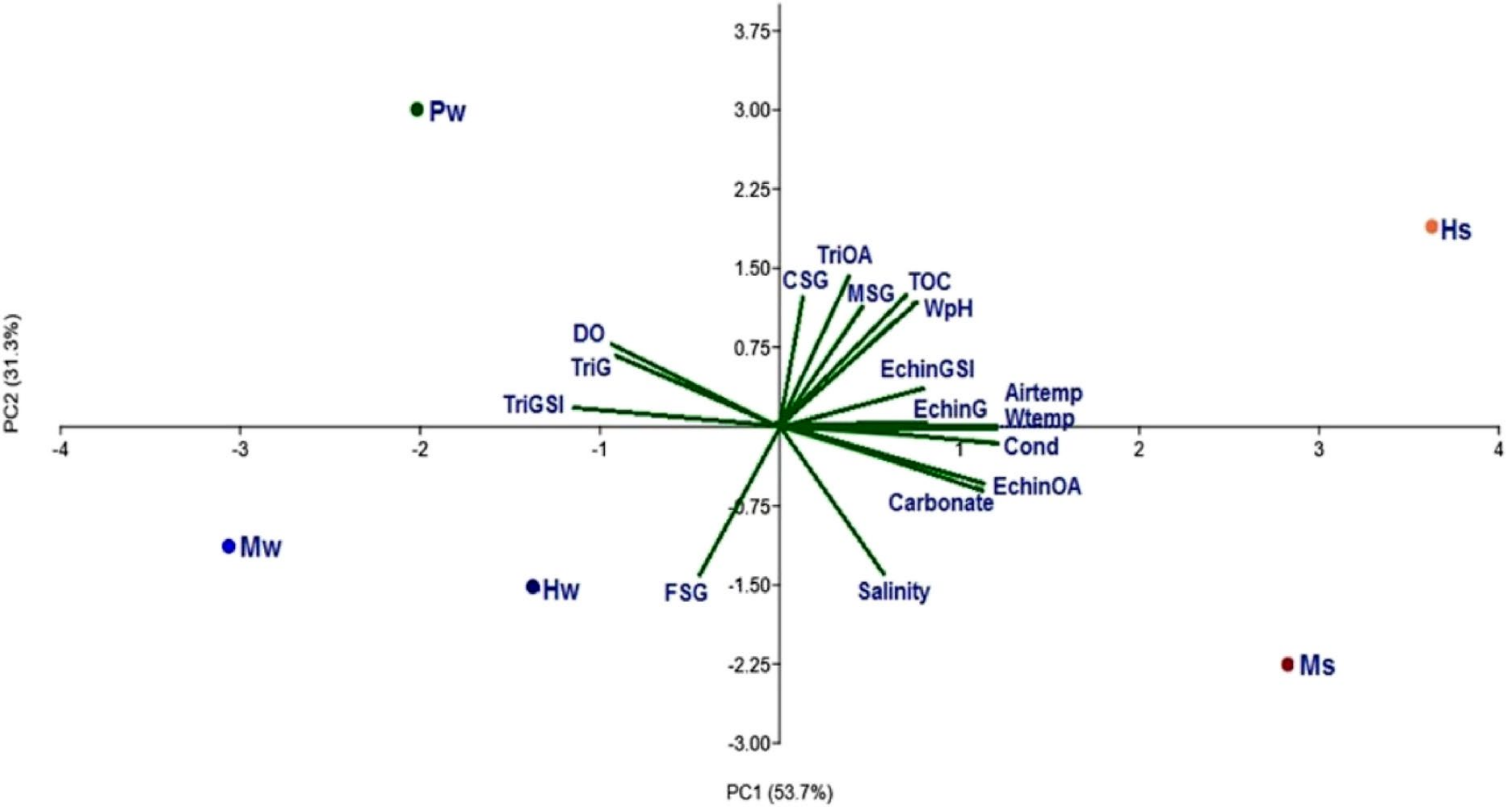
Biplot of principal component analysis (PCA) of environmental variables and reproductive activity of *Echinometra mathaei* (e) and *Tridacna squamosa* (t) recorded for different sampling groups*.* Environmental variables: Airtemp, air temperature; Wtemp, water temperature; WpH, water hydrogen ion concentration; Cond, conductivity; Salinity, water salinity; Carbonate, sediment carbonate content; TOC, sediment total organic matter content; DO, dissolved oxygen; CSG, coarse sediment group; MSG, medium sediment group; FSG, fine sediment group. Reproductive activity: Echin,* E. mathaei*; Tri, *T. squamosa*; GSI, gonadosomatic index; G, gamete percentage; OA, ovum area

According to PCA, the PC axis 1 clarified 53.7% of the total variation of environmental variables and reproductive activity of the studied species, separating summer samples (Ms and Hs) on the positive side of the axis from winter samples (Mw, Hw, and Pw) on the negative side of the axis. PC axis 2 (31.3%) separated sampling groups into Hs and Pw on the positive side and Hw, Ms, and Mw on the negative side (Fig. 10). The GSI, percentage of gametes, and ovum area of *E. mathaei* were positively correlated with air temperature and water temperature, conductivity, and carbonate content and negatively correlated with dissolved oxygen and FSG; GSI and percentage of gametes of *T. squamosa* showed a reverse effect of these variables. The ovum area of *T. squamosa* was positively correlated with CSG, MSG, TOC, WpH, water, and temperature and negatively correlated with FSG, salinity, and carbonate content (Fig. 10).

## Discussion

The results of gametes ratio show variations between *E. mathaei* and *T. squamosa*. In *E. mathaei*, the percentages of male gametes were dominant over female gametes. Siddique and Ayub (2019) illustrated that the dominancy of one sex over the other may be related to differences in growth, mortality, and gonadal maturation. On the other hand *T. squamosa* show seasonal variations in gamete ratio. As female gametes were dominant over male gametes at Hamraween during both seasons, male gametes were dominant over female gametes at Sedy Malek during summer. These differences in *T. squamosa* may be due to its nature as a protandric hermaphrodite species. Previous studies showed that transformation from one gamete state to another is a continual process in bivalves and is influenced by environmental factors (Cano et al., 1997; Menoud et al., 2016).

The results show that the GSI in *T. squamosa* is higher than that in *E. mathaei*. This may be due to the fact that the digestive gland of *T. squamosa* is attached to the gonad and cannot be separated easily (Menoud et al., 2016), and therefore, its weight is added to the weight of the gonads. Many authors have warned against relying on the GSI alone because it provides little hint of the development of the gametes themselves. They recommend using histologic methods in combination with GSI (Mercier & Hamel, 2009; Nichols et al., 1985). For example, during gametogenesis, when nutritive tissue is utilized to synthesize the gametes, the GSI index may remain unchanged, despite an increase in actual gametogenesis (Nichols & Barker, 1984). Therefore, the present study used both the GSI and histologic sections of the investigated species.

The results show that *E. mathaei* has active reproductive behavior during the summer season. This rhythm is also recorded for the same species in different locations (Bronstein & Loya, 2015; Keshavarz et al., 2017; Muthiga & Jaccarini, 2005). These studies obtained their results according to GSI, whereas the present study confirmed them by histologic analyses. According to the gonad index, Bronstein et al. (2016) claimed that *Diadema setosum* has two main spawning events. However, they found that histological analysis shows that this is not the case and that the second peak of gonad index values actually represents recuperating individuals. Keshavarz et al. (2017) compared spawning months of *E. mathaei* in different locations. Most of these studies indicated that *E. mathaei* spawned from March to September. In contradiction to this, Siddique and Ayub (2019) reported that *E. mathaei* spawned from September to January. Pearse (1969) revealed significant differences in the reproductive strategies of Echinometra populations across the regions. In contrast to continuous spawning in the northern Red Sea, he noted a single spawning period (July to September) in the Gulf of Suez. He suggested that the management of the reproductive periodicities depends on both a critical minimum temperature and a critical minimum quantity of nutritional reserves.

Byrne et al. (1997) showed that seasonality in ecosystems increases gradually from the tropics to the Polar Regions, which has important effects on the reproduction of echinoderms. Tropical marine animals breed throughout the year, whereas those living outside the tropical regions show seasonality in breeding activities (Giese & Pearse, 1974). Siddique and Ayub (2019) showed that tropical species such as *Echinometra* and *Diadema* species when inhabiting temperate or subtropical environments are adapted to reproduce during limited periods of the year (Alsaffar & Lone, 2000; Hernández et al., 2006). This led to the conclusion that the reproductive activities of animals inhibiting temperate or subtropical regions are affected by temperature fluctuations via month’s variation.

In the present study, based on gonad histologic analysis, the individuals of *T. squamosa* showed variability of maturation stages in the gonads. High variability of maturation stages at the intragonadal scale in bivalves was recorded in previous studies (Pouvreau et al., 2000; Delgado & Pérez-Camacho, 2007; Menoud et al., 2016). The current results indicate that male and female maturation of *T. squamosa* was an alternative between oogenesis and spermatogenesis. This asynchronous maturity of male and female tissues was recorded for other *Tridacna* species: *T. gigas* (Nash et al., 1988) and *T. maxima* (Menoud et al., 2016).

Menoud et al. (2016) concluded that the maturation of *Tridacna* can be detected by the shape and size of the ovum. In general, the ovum size of *T. squamosa* indicated that maturation occurs during the winter. In contrast, the maturation of *T. squamosa* ova was recorded at El-Hamrawen during the summer season. This difference may be related to the availability of food at El-Hamrawen where the availability of nutrition since phosphate particles spread and fall to the sea at this site. Mercier and Hamel (2009) showed that in most marine species, the effects of nutrition on reproduction are due to the metabolic costs of gamete synthesis rather than the timing of the breeding season. Hold et al. (2013) showed that the reproductive success of bivalve molluscs differed among sites and habitats on a small scale. They found that *Pecten maximus* showed variation in gonadal condition over a distance of less than 5 km.

Various factors, mainly environmental factors such as temperature, light, salinity, availability of food, pH, or even the population density in some studies (Keshavarz et al., 2017), affect the reproductive timing of *Echinometra*. The present results showed that the reproductive activity and gonadal maturation of *E. mathaei* were positively correlated with air temperature and water temperature, conductivity, and carbonate content and negatively correlated with dissolved oxygen and FSG. This may be due to the higher values of GSI, percentage of gametes, and ovum area in *E. mathaei* during the summer season, which is characterized by relatively high temperatures. Rather than the direct effect of temperature, its indirect effects can appear in its positive correlation with water conductivity (Dauphinee & Klein, 1977) and negative correlation with dissolved oxygen (Manasrah et al., 2006). Previous studies indicated that temperature is the key environmental factor influencing the seasonal reproductive pattern of *E. mathaei* (Alsaffar & Lone, 2000; Muthiga & Jaccarini, 2005). A positive correlation effect of seawater temperature on another sea urchin has been recorded (Pearse et al., 1986 “*Strongylocentrotus purpuratus*”; Walker & Lesser, 1998 “*Strongylocentrotus droebachiensis*”; Pérez et al., 2010 “*Loxechinus albus*”; Brogger et al., 2010 “*Arbacia dufresnii*”).

Unlike *E. mathaei*, the reproductive activity and gonadal maturation of *T. squamosa* were positively correlated with dissolved oxygen and negatively correlated with water temperature, conductivity, and carbonate. This can explain the maturation of *T. squamosa* during the winter, which is promoted by the relatively low water temperature and high dissolved oxygen. Pouvreau et al. (2000) proposed that the low disparity in temperature between the seasons in the tropics encourages continuous gametogenesis for most tropical bivalves. Previous studies suggested that the reproductive activity of giant clams is associated with temperature changes (Gilbert et al., 2006; Van Wynsberge et al., 2017). Van Wynsberge et al. (2017) found that, during the seasonal decline in temperature, the values of GSI became more variable. They concluded that the reproductive activities of the giant clam *T. maxima* were timed to a decrease in water temperature.

## Conclusions

The present study provides new results for two bioindicators of the marine benthos, *E. mathaei* and *T. squamosa*, which will help future studies to understand the regeneration of these populations and of other benthos with similar reproductive strategies as well. Both species showed spatial, seasonal, and species-specific variations of gonadal development and reproductive activities. *E. mathaei* showed more active reproductive behavior in the summer than in the winter; the reproductive behavior of *T. squamosa* was active almost all the year, especially during the winter. The results support the hypothesis that water temperature is a vital factor that affects the reproductive activity of marine organisms. Therefore, changes in seawater temperature could affect reproductive timing and the renewal and stability of these species. This study concluded that changes in temperature brought on by global warming may have considerable effects on coastal marine ecosystems.

### Supplementary Information

Below is the link to the electronic supplementary material.

Supplementary file1 (PDF 1234 KB)

## Data Availability

The datasets of the current study are available from the corresponding author on reasonable request.
