# Supplementary material for: Influences of habitat and seasonal changes on gonadal maturation of Echinometra mathaei (Echinodermata: Echinoidea) and Tridacna squamosa (Mollusca: Bivalvia) in the Red Sea, Egypt
Source: Environ Monit Assess. 2023 Aug 24;195(9):1088. doi: 10.1007/s10661-023-11713-9 (PMC10449673; doi:10.1007/s10661-023-11713-9)
Supplement: Supplementary file 1 — Supplementary file1 (PDF 1234 KB) [file 10661_2023_11713_MOESM1_ESM.pdf]

Supplementary Materials for

Influences of habitat and seasonal changes on gonadal maturation of *Echinometra mathaei* (Echinodermata: Echinoidea) and *Tridacna squamosa* (Mollusca: Bivalvia) in the Red Sea, Egypt

Samaa G. El-Sokkary[a], Khaleid F. Abd El-Wakeil[a*], Ahmad H. Obuid-Allah[a] and Mohsen Y. Omer[b]

[a] *Zoology and Entomology Department, Faculty of Science, Assiut University, Egypt*
[b] *National Institute of Oceanography and Fisheries (NIOF), Red Sea Branch, Egypt*

[*] *Corresponding author:* kfwakeil@yahoo.com, kfwakeil@aun.edu.eg

This file illustrates how the ImageJ program was used to calculate the ratio of gametes to nutritive cells and ovum area for the taken photos.

The hematoxylin and eosin stained of gonads sections were examined by light microscope (Olympus CHT). Four sections were selected for each animal samples. Three photographs at different locations from selected four sections per collected animals were taken by XCAM1080PHA camera. ImageJ program was used to calculate the ratio of gametes to nutritive cells and ovum area for the taken photos as following.

The ratio of gametes to nutritive cells measurement

1- Set scale for the photo scale bar of the photo.

2- Create grids by using crosses which an area per point equal 20000 µm2

3- Count gamete if the Crosse is in front of the gamete and count the nutritive when it is in front of the nutritive cell (Fig. 1.2.3).

4- The counted gametes and nutritive cells were used to calculate the percentage of gamete percentage.

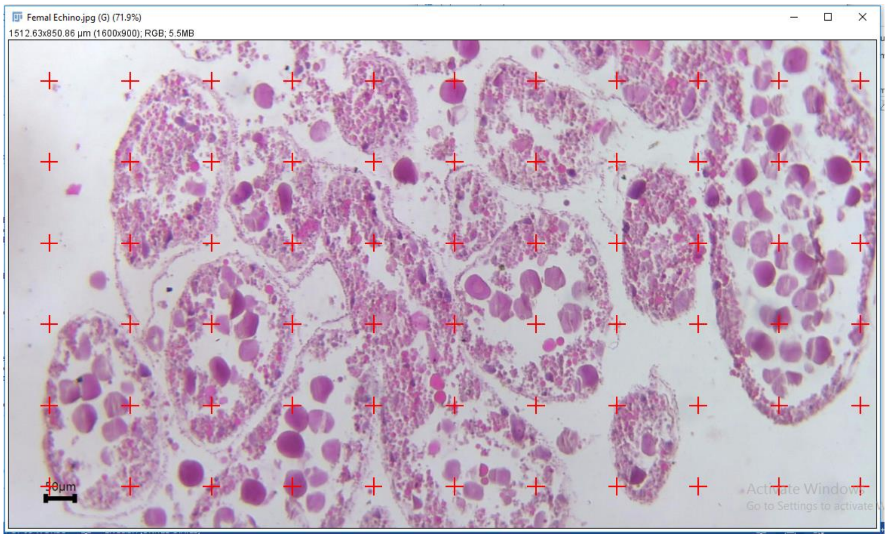

Fig. 1.

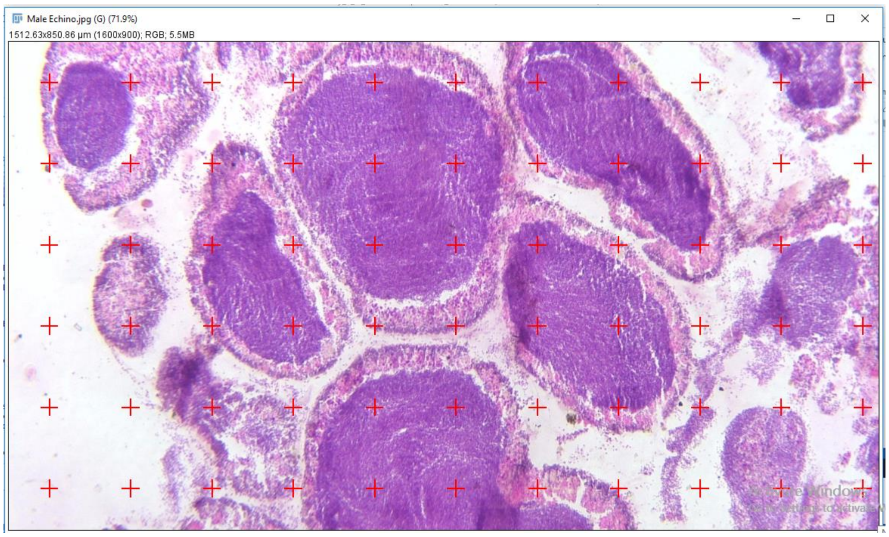

Fig. 2.

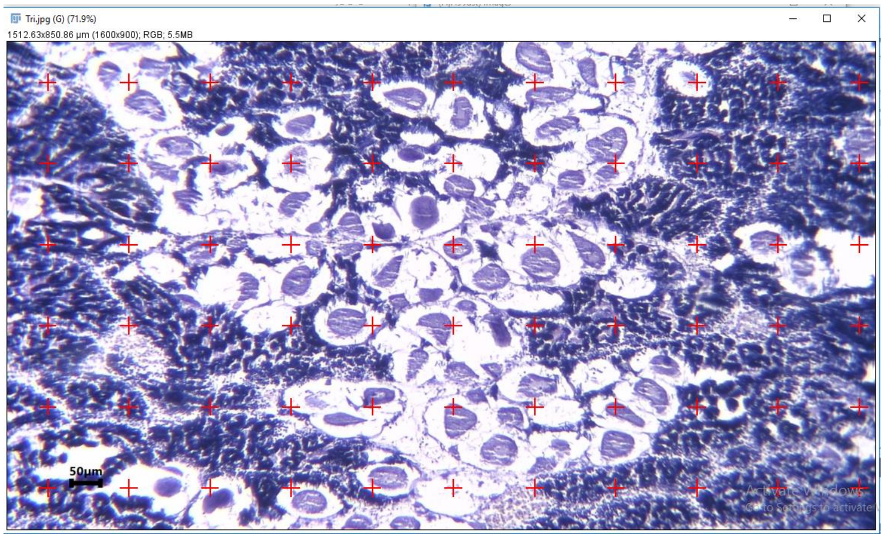

Fig. 3.

Ovum area measurement

1- Set scale for the photo scale bar of the photo.

2- 15 oocytes were selected randomly from the female photographs.

3- For each oocyte, the longest and shortest diameters were measured (Fig. 4)

4- The measured Longest and shortest diameters were used to calculate the ovum area as an oval shape.

$$\text{Ovum area} = Pi * \frac{\text{the longest diameter}}{2} * \frac{\text{the shortest diameter}}{2}$$

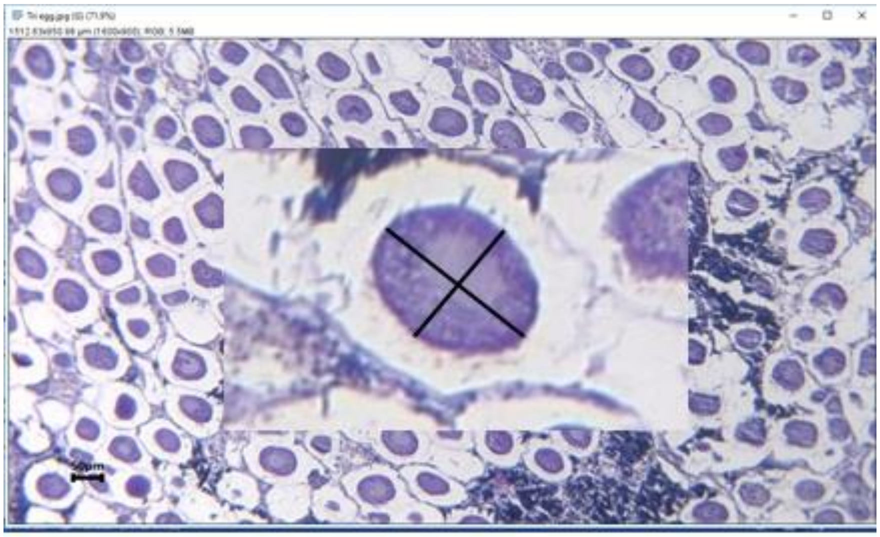

Fig. 4. Illustrative example of the longest and shortest diameters of oocyte.
